# Fusarium Head Blight From a Microbiome Perspective

**DOI:** 10.3389/fmicb.2021.628373

**Published:** 2021-03-01

**Authors:** Ida Karlsson, Paula Persson, Hanna Friberg

**Affiliations:** ^1^Department of Crop Production Ecology, Swedish University of Agricultural Sciences, Uppsala, Sweden; ^2^Department of Forest Mycology and Plant Pathology, Swedish University of Agricultural Sciences, Uppsala, Sweden

**Keywords:** Fusarium head blight (FHB), scab, Fusarium crown rot (FCR), cereals, pathogen–pathogen interactions, pathogen–microbe interactions

## Abstract

The fungal genus *Fusarium* causes several diseases in cereals, including Fusarium head blight (FHB). A number of *Fusarium* species are involved in disease development and mycotoxin contamination. Lately, the importance of interactions between plant pathogens and the plant microbiome has been increasingly recognized. In this review, we address the significance of the cereal microbiome for the development of *Fusarium*-related diseases. *Fusarium* fungi may interact with the host microbiome at multiple stages during their life cycles and in different plant organs including roots, stems, leaves, heads, and crop residues. There are interactions between *Fusarium* and other fungi and bacteria as well as among *Fusarium* species. Recent studies have provided a map of the cereal microbiome and revealed how different biotic and abiotic factors drive microbiome assembly. This review synthesizes the current understanding of the cereal microbiome and the implications for *Fusarium* infection, FHB development, disease control, and mycotoxin contamination. Although annual and regional variations in predominant species are significant, much research has focused on *Fusarium graminearum*. Surveying the total *Fusarium* community in environmental samples is now facilitated with novel metabarcoding methods. Further, infection with multiple *Fusarium* species has been shown to affect disease severity and mycotoxin contamination. A better mechanistic understanding of such multiple infections is necessary to be able to predict the outcome in terms of disease development and mycotoxin production. The knowledge on the composition of the cereal microbiome under different environmental and agricultural conditions is growing. Future studies are needed to clearly link microbiome structure to *Fusarium* suppression in order to develop novel disease management strategies for example based on conservation biological control approaches.

## Introduction

Fusarium head blight (FHB) is one of the most important cereal diseases worldwide. The disease results in reduced yields and mycotoxin contamination of the grain. Several different *Fusarium* species are associated with FHB. The predominant species varies with region and climate. Globally, *Fusarium graminearum* is considered the predominant causal species of FHB (Starkey et al., 2007; Summerell et al., 2010). In Europe, *F. graminearum*, *Fusarium culmorum*, *Fusarium poae*, and *Fusarium avenaceum* are considered to be most common (Xu et al., 2008; Becher et al., 2013). Often, several *Fusarium* species are present simultaneously, and may interact with each other, influencing disease development and mycotoxin production. In this review, we use the term “FHB species complex” to refer to the *Fusarium* species associated to FHB and mycotoxin contamination in cereals.

*Fusarium* fungi can in addition to FHB cause other diseases in cereals during their life cycle including seedling blight, root rot and *Fusarium* crown rot (FCR; Parry et al., 1995; Kazan et al., 2012). Many *Fusarium* species are capable of producing mycotoxins, in some cases also in absence of severe disease symptoms. The mycotoxins of greatest concern include the trichothecenes deoxynivalenol (DON), nivalenol, and HT2/T2, and the oestrogenic mycotoxin zearalenone (ZEA; Bottalico and Perrone, 2002). *F*. *graminearum* and *F*. *culmorum* are important producers of DON, ZEA, and nivalenol, the latter may also be produced by *F*. *poae* (Bottalico and Perrone, 2002). *Fusarium langsethiae* and *Fusarium sporotrichioides* are producers of the HT2 and T2 toxins (Thrane et al., 2004). Novel analytical methods has made it possible to analyze many mycotoxins simultaneously. This has drawn the attention to so called “emerging” *Fusarium* toxins, where knowledge on toxicity is limited (Fraeyman et al., 2017). Examples include enniatins and moniliformin which may be produced by *F*. *avenaceum* (Morrison et al., 2002). For a recent overview of mycotoxin-producing *Fusarium* species see Venkatesh and Keller (2019). To protect consumer health, legal limits have been set in many countries for maximum mycotoxin content in unprocessed grain and foodstuffs. In Europe, DON, ZEA, and fumonisins are regulated (European Commission, 2006). The legislation will likely cover more mycotoxins in the future such as HT2/T2 (Knutsen et al., 2017). Another topic of discussion is the existence of conjugated toxin forms, formed when metabolized by the plant, sometimes called “masked mycotoxins” (Zhang et al., 2020). *Fusarium* mycotoxins are a concern for human and animal health and the economic consequences may be severe when contaminated grain cannot be used for food or feed.

Certain risk factors for FHB are well-known. Weather conditions at flowering is one of the most important factors for infection along with certain cropping practices such as reduced tillage and maize as a preceding crop to cereals (Dill-Macky and Jones, 2000; Beyer et al., 2006; Edwards and Jennings, 2018). Inconsistent results have been obtained for nitrogen fertilization which has been linked both to increased and decreased FHB symptoms and mycotoxin accumulation (Lemmens et al., 2004; Heier et al., 2005; Hofer et al., 2016; Zetzsche et al., 2020). Effective chemical control is often difficult to achieve as it is dependent on optimal timing of the fungicide application (Wegulo et al., 2015). However, interactions with naturally occurring microorganisms is less well-understood and is the focus of this review.

Plants are increasingly seen as holobionts where plant-associated microbiota play an important role for plant fitness (Vandenkoornhuyse et al., 2015). Plants harbor complex microbial communities both below- and aboveground. Advances in this research area are deeply influenced by the development of metabarcoding approaches and omics to study phytobiomes (Rastogi et al., 2013). In plant pathology, these tools have opened up new possibilities to understand for instance, disease suppressive soils or to improve biocontrol applications (Massart et al., 2015; Schlatter et al., 2017). The existence of specific beneficial microbial strains has been known for decades. These may have both direct and indirect effects on the plant including induced systemic resistance, production of secondary metabolites, hormones or through nutrient effects (van Loon, 2007). Plant-associated microorganisms may also facilitate the infection by plant pathogens (Dewey et al., 1999). Recently, evidence is gathering that it is not solely the individual strains that are important for plant health but also the microbial community both aboveground (Ritpitakphong et al., 2016; Zahn and Amend, 2017) and belowground (Hol et al., 2015; Hu et al., 2016).

In this review, we highlight FHB and associated diseases from a microbiome perspective. In the first part, starting with a review of the FHB disease cycle, a holistic view of *Fusarium* spp. in cereals is taken. Novel methods to survey the *Fusarium* community composition are described. Next, interactions between different *Fusarium* species and the implications for FHB disease development and mycotoxin contamination are reviewed. In the second part, we synthesize recent findings concerning the cereal microbiome in different plant organs. Finally, the importance of the microbiome for FHB and the potential for prevention or control of the disease and mycotoxin accumulation is discussed.

## FHB Disease Cycle

*Fusarium* species differ in their biology, for instance the types of spores they produce. These characteristics will influence how they are spread within agroecosystems. *Fusarium* species produce asexual conidia and several species, for example *F. graminearum* and *F. avenaceum*, also have sexually produced ascospores. The sexual stages were previously described by their teleomorph names in the genus *Gibberella* (Geiser et al., 2013). Some species also produce chlamydospores with thicker cell walls with the possibility to survive in the soil, such as *F. culmorum* and *F. graminearum* (Leslie and Summerell, 2006). Spore dissemination is a critical step for the fungus to colonize new plant parts. The sexual ascospores are known to be released by active mechanisms of the perithecia while conidia, the asexual spores, are dependent on wind or rain for liberation (Trail et al., 2005). Macroconidia are reported to be splash-dispersed short distances within the canopy while ascospores can be transported longer distances by wind (Keller et al., 2014). The spore type might also be of importance for disease development. For instance, ascospores of *F. graminearum* were shown to be less effective in causing FHB and FCR than conidia, although the difference in FHB severity between the two spore types was small (Mitter et al., 2006).

There has been a long debate on whether local or air-transported spores are the main inoculum source of FHB epidemics caused by *F. graminearum* (Keller et al., 2014). In aerial samples 60 m above ground level, twice as many ascospores compared to macroconidia were reported by Maldonado-Ramirez et al. (2005). They showed that viable *F. graminearum* spores were abundant in the air every hour of the day and night indicating that that the source of these spores was likely several kilometers away. Transport of *F. graminearum* in the atmosphere may be responsible for initiating disease many kilometers from the inoculum source. Thus, in addition to the presence of the pathogen at the field level, atmospheric inoculum needs to be taken into account when evaluating the risk of FHB.

Contaminated crop residues are an important *Fusarium* inoculum source as they allow for saprotrophic survival of the pathogen (Leplat et al., 2012). Maize is the preceding crop associated with highest risk of FHB since it is a good host for *Fusarium* spp. and produce large amounts of residues (Dill-Macky and Jones, 2000; Tillmann et al., 2017). Several of the species that are important for FHB can survive on crop residues. This applies for example to *F. graminearum*, *F. culmorum*, *F. avenaceum*, *F. poae*, and *F. sporotrichioides*. For *F. langsethiae*, the role of crop residues for pathogen survival is less clear, although studies have shown a correlation between residue retention in the field and increased disease (Hofgaard et al., 2016b). Due to the important role of crop residues, a well-planned cropping sequence and tillage strategy are important parts of crop protection strategies against FHB. When soils are plowed and residues are buried in the soil, this means that the residues are no longer in close contact with the crop canopy. It also means that the residues are exposed to soil organisms in an environment that stimulates microbial growth and microbial decomposition of the residues.

Spore production varies between *Fusarium* species and over the growing season. By analyzing *Fusarium* DNA from spore traps in Norway, Hofgaard et al. (2016b), observed that *F. avenaceum* had a less marked peak of spore dispersal than *F. graminearum*, but had a relatively stable dispersal of spores from shortly before heading until harvest time. Hellin et al. (2018) showed from studies in Belgium that inoculum of *F. graminearum* was present not only during the flowering period but also throughout the year with large amounts detected late in the season. This is also confirmed by Swedish studies showing that *F. graminearum* perithecia and ascospores are produced during the entire growing season on artificially inoculated maize and wheat straw in the field (Persson and Bötker, 2014). This indicates that aerial *Fusarium* spores are present and could potentially infect cereal crops during the entire growing season.

It is well known that the most important window for head infection by *Fusarium* species causing FHB is during cereal anthesis (Hooker et al., 2002) whereas the importance of aerial spore loads after anthesis is unclear. Siou et al. (2014) showed that anthesis is the critical window for FHB infection of wheat but also that later infections may lead to a significant development of the fungus, along with the accumulation of toxins in the kernels, although with limited symptom development. The authors claim that late infections may lead to significant toxin levels and might also have consequences for the seed production with viable but *Fusarium*-contaminated seeds. It has also been shown in barley and oats that infection can occur after anthesis (Yoshida et al., 2007; Tekle et al., 2012). Interestingly, the effect of infection time varies with different *Fusarium* species (Beccari et al., 2019).

**FIGURE 1:**
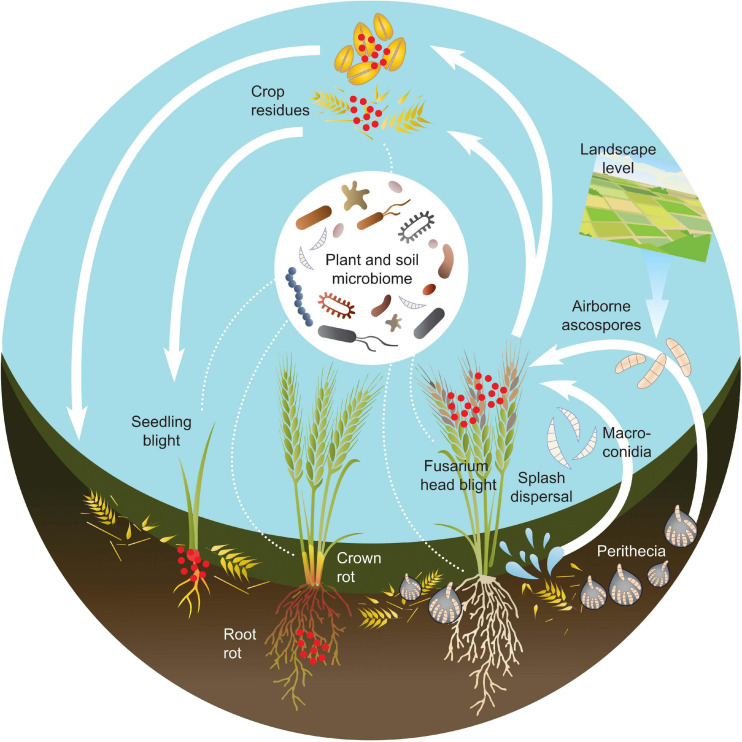
Schematic figure over the disease cycle and microbiome interactions of *Fusarium*-related diseases in cereals, based on the life cycle of *F. graminearum*. The red dots represent *Fusarium* inoculum. *Fusarium* fungi can cause several diseases during the development of cereal crops including: seedling blight, Fusarium root rot, Fusarium crown rot and Fusarium head blight. *Fusarium* inoculum can originate both from the field itself, if present in crop residues or soil. It can also be introduced by infected seed or by deposition of spores from other areas. Other microorganisms are present both in the soil and on the plant. The microbiome structure varies depending on both plant organ, plant developmental stage, environmental and agricultural factors. *Fusarium* species are in constant contact with various microbiomes throughout the entire disease cycle.

The inoculum for FHB may thus come both from sources within the field or from aerial spore depositions originating from outside the field, and occur at different growth stages. This means that the potential for interactions between *Fusarium* species and the plant microbiome will vary over time and with plant organ (Figure 1).

## Interactions Within the FHB Species Complex

### FHB Species Complex

The genus *Fusarium* is diverse, containing species with various ecological characteristics. Several species are commonly found associated with plant tissue, with influence ranging from severely pathogenic to highly beneficial as biological control agents. Studies on the abundance and distribution of species associated with cereal crops have mainly focused on pathogens causing either FHB or FCR or on species that cause problems due to their mycotoxin production. There is a considerable spatiotemporal variation in the presence and relative abundance of species in association with FHB on a specific host plant. This variation is determined by climatic factors, especially temperature and moisture (Vogelgsang et al., 2017), crop species (Kosiak et al., 2003; Schöneberg et al., 2016, 2018) plant part (Tillmann et al., 2017), region and year (Yli-Mattila, 2010; Sundheim et al., 2013; Hofgaard et al., 2016b; Vogelgsang et al., 2017), and by cultural practices favoring certain species (Schöneberg et al., 2018). There may also be geographical restrictions to species distributions (Yli-Mattila, 2010).

A broad range of *Fusarium* species have been associated with FHB in cereals. In addition, the pathogens *Microdochium nivale* and *Microdochium majus* are commonly included in the concept of FHB as these species can cause head blight symptoms (Xu et al., 2005; Oerke et al., 2010; Nielsen et al., 2011). There is a large variability in the importance of the different species for FHB disease severity and mycotoxin contamination. Some species cause both severe disease symptoms and mycotoxin contamination of grains, for instance *F. graminearum* and *F. culmorum*. In contrast, species within the genus *Microdochium* are not known to produce any mycotoxins (Brennan et al., 2005; Gavrilova et al., 2020). Other species, such as *F. langsethiae* can produce potent mycotoxins while causing limited symptoms (Imathiu et al., 2013). Since it is not easy to make a clear delimitation of the FHB species complex based on pathogenicity or toxigenicity, we here include all *Fusarium* species associated with cereals together with *M. nivale* and *M. majus* in the concept.

The number of *Fusarium* species associated with cereal grain has in the literature been estimated to around 15 species, including *M. nivale* and *M. majus* (Bottalico and Perrone, 2002; Liddell, 2003). We reviewed the recent literature to identify the range of *Fusarium* species occurring in cereals in Europe, identifying 17 species of which many were only reported sporadically and/or in low abundance (Table 1). A literature review identified that *F. graminearum, F. culmorum, F. avenaceum*, and *F. poae* had the highest incidence in Europe (Becher et al., 2013). The species with the highest incidence may differ from those with the highest abundance. For instance, *F. avenaceum* had the highest incidence, while *F. graminearum* had the highest relative abundance in wheat samples in Sweden (Karlsson et al., 2017a).

**TABLE 1:** Diversity* of *Fusarium* and *Microdochium* species reported from cereal grain in Europe.

Species	References
*F. acuminatum*	7,8
*F. avenaceum*	1,2,3,4,5,6,7,8
*F. chlamydosporum*	4
*F. crookwellense* (*F. cerealis*)	2,4,5,6
*F. culmorum*	1,2,3,4,5,6,7,8
*F. dimerum*	6
*F. equiseti*	2,4,5,6,8
*F. graminearum*	1,2,3,5,6,7,8
*F. langsethiae*	3,5,6,7,8
*F. lateritium*	7
*F. proliferatum*	4
*F. poae*	1,2,3,4,5,6,7,8
*F. sambucinum*	4
*F. semitectum*	4
*F. sporotrichioides*	2,3,4,5,6,7,8
*F. subglutinans*	4
*F. tricinctum*	2,3,4,5,6,7,8
*F. verticilloides*	4
*M. majus*	1,2,3**,4**
*M. nivale*	1,3**,4**

*Reference, location, cereal species, and identification method:*

*1 Xu et al. (2005): Hungary, Italy, Ireland, UK, wheat, PCR*

*2 Oerke et al. (2010): Germany, wheat, isolation*

*3 Nielsen et al. (2011): Denmark, wheat, barley, oat, triticale, rye, qPCR*

*4 Infantino et al. (2012): Italy, durum and bread wheat, freezing blotter method*

*5 Sundheim et al. (2013): Norway, wheat, barley, oat, isolation*

*6 Schöneberg et al. (2016): Switzerland, barley, isolation*

*7 Karlsson et al. (2017a): Sweden, wheat, metabarcoding*

8 Beccari et al. (2018b): Italy, barley, isolation and molecular identification

*There are many more studies that have assessed the occurrence of different *Fusarium* species in Europe. Here we iteratively added references that extended the list of reported species until no more species could be added. Therefore, many studies targeting only a handful of species were excluded. **These studies did not separate between the two *Microdochium* species.

Previously, *F. culmorum* was considered to be more prevalent in Northern Europe and *F. graminearum* to be more prevalent in Southern Europe (Bottalico and Perrone, 2002). A shift in the predominant species from *F. culmorum* to *F. graminearum* has been described in Northern Europe in recent decades (Jennings et al., 2004; Waalwijk et al., 2004; Nielsen et al., 2011; Bilska et al., 2018; Hofer et al., 2019). It has been suggested that the shift towards *F. graminearum* could be due to increased practice of reduced tillage and maize cultivation, or to climatic factors (Nielsen et al., 2011; Parikka et al., 2012). In Italy, a shift in predominant species from *F*. *graminearum* to *F*. *poae* has been observed in recent years (Beccari et al., 2017). Similar observations have been made in Canada, and it is speculated that *F*. *poae* is favored by dry conditions compared to *F*. *graminearum* (Valverde-Bogantes et al., 2019).

A shift in the population structure of *F. graminearum* from 15ADON chemotypes towards more toxigenic 3ADON chemotypes has been observed in North America (Ward et al., 2008), and recently a third *F. graminearum* chemotype – NX-2 – was identified producing a novel type A trichothecene (Kelly and Ward, 2018). In Europe, the 15ADON chemotype dominates in Southern Europe and the 3ADON in Northern Europe, which could be associated with the more frequent oat cultivation in Northern Europe (Aamot et al., 2015; Pasquali et al., 2016).

The situation with a complex of species causing or being associated with the disease rather than a single species, makes it more difficult to understand and manage FHB. Species within the FHB complex may have differences in their sensitivity to fungicides, leading to inconsistency in effects of chemical control (Pirgozliev et al., 2003). This also has implications when breeding for FHB resistance, as crop cultivars may differ in susceptibility, with lower sensitivity to one *Fusarium* species and not necessarily to another (Vogelgsang et al., 2008). There are, however, also examples of cultivars that show resistance against several *Fusarium* species (Linkmeyer et al., 2013).

Several *Fusarium* species can infect roots and crowns of cereals and cause FCR. The species are partly overlapping with those associated with FHB. It has been suggested that infected stem bases can be a source of inoculum for head infections (Parry et al., 1995). Tillmann et al. (2017) used isolation and identified *F. culmorum*, *Fusarium equiseti*, and *Fusarium tricinctum*, and to a lesser extent *F. graminearum*, *Fusarium oxysporum*, and *F. avenaceum* on stem bases in Germany. In their study, the colonization frequency of the different *Fusarium* spp. differed with crop rotation sequence. Shikur Gebremariam et al. (2018) isolated *Fusarium* fungi from the crown of wheat plants (bread wheat and durum wheat) from different regions of Turkey, and found 17 species, of which six were found to cause crown rot (*F. avenaceum, F. culmorum, F. graminearum, Fusarium hostae, Fusarium pseudograminearum*, and *Fusarium redolens*) whereas *F. oxysporum, F. equiseti, Fusarium solani, Fusarium incarnatum* (syn. *Fusarium semitectum*), *Fusarium reticulatum* (syn. *Fusarium heterosporum*), *Fusarium flocciferum, Fusarium tricinctum, Fusarium brachygibbosum, Fusarium torulosum, Fusarium acuminatum, and Fusarium proliferatum* were found not to cause any symptoms.

Although the FCR pathogens *F. graminearum*, *F. culmorum*, or *F. pseudograminearum* can grow systemically from infected crowns, they do not appear to colonize as far as to the head. However, DON is water-soluble and may be translocated to the heads without fungal growth indicating that FCR could be a potential additional source of DON. Beccari et al. (2018a) observed low amounts of DON translocated to the heads, but higher concentrations in stems. *F. graminearum* can also colonize the stem downwards from the heads (Guenther and Trail, 2005). An interesting observation made recently is that significant levels of DON originating from *F. graminearum* infections, was found in both wheat, barley and oat straw aimed for animal feed (Häggblom and Nordkvist, 2015). Late infection at least 20 days after anthesis caused toxin contamination of grain without clear disease symptoms on the spike (Siou et al., 2014). This suggests that control strategies that cover the late as well as early stage of grain development may be considered as an effective measure to reduce the final level of mycotoxins and may also reduce the risks for toxin contamination of the straw.

### Methods Targeting the FHB Species Complex

The frequent occurrence of several *Fusarium* species in cereals makes monitoring more challenging. Isolation techniques for characterization of fungal communities are heavily biased toward fast-growing species and those favored by the growth medium and temperatures used for isolation. Until recently, molecular methods relied mostly on the identification of one species at a time. For example, Nicolaisen et al. (2009) presented individual real-time PCR assays for quantification of 11 *Fusarium* species. There are also examples of multiplex PCR for simultaneous molecular quantification of several *Fusarium* species, but these are limited to a handful of species (Bluhm et al., 2004; Yli-Mattila et al., 2008).

Metabarcoding is the current state-of-the-art technology for characterizing fungal communities. Recently, several metabarcoding approaches have been developed to characterize *Fusarium* communities in environmental samples (Table 2). Although some information about *Fusarium* can be obtained using metabarcoding targeting the fungal internal transcribed spacer (ITS), this gene does not provide species-level resolution for *Fusarium* (Nicolaisen et al., 2014). *Fusarium* species are also known to carry non-orthologous copies of the ITS, which may hamper correct diversity estimation (O’Donnell and Cigelnik, 1997). In addition to the choice of marker gene, there are several sequencing platforms available. Early platforms included 454 sequencing which was outcompeted by techniques with higher output but shorter read length such as Illumina MiSeq. More recently, longer read lengths up to several kbp can be achieved with the so called third generation technologies such as the PacBio platform (Tedersoo et al., 2018; van Dijk et al., 2018). The major advantage of using longer read lengths is that it can improve taxonomic resolution (Singer et al., 2016).

**TABLE 2:** Metabarcoding approaches targeting *Fusarium* communities in environmental samples.

References	Target gene	Amplicon length (bp)	*Fusarium*-specific	Sequencing platform	Substrate
LeBlanc et al. (2015)	*RPB2*	730	no	454	rhizosphere
Walder et al. (2017)	ITS-LSU	1600	no	PacBio SMRT	wheat, cover crops and maize
Karlsson et al. (2016)	TEF-1α	550-600	yes	454	wheat kernels, soil
Boutigny et al. (2019)	TEF-1α	640	yes	Illumina Miseq 2x250 bp	cereal grain
Cobo-Díaz et al. (2019)	TEF-1α	430	yes	Illumina Miseq 2x300 bp	maize residues, soil

*RPB2, DNA-directed RNA polymerase II second largest subunit; ITS-LSU, internal transcribed spacer region and large subunit of the rRNA; TEF-1α, translation elongation factor 1-alpha.*

Several combinations of marker genes and sequencing platforms have been used to characterize *Fusarium* communities (Table 2). LeBlanc et al. (2015) used primers targeting the partial DNA-directed RNA polymerase II second largest subunit (*RPB2*), enriching for taxa in the Sordariomycetes, in a metabarcoding study targeting *Fusarium* species in the rhizosphere, identifying about 13% of sequences as *Fusarium*. The first approach to use *Fusarium*-specific primers, targeted the elongation factor (*TEF-1α*) which is single-copy in *Fusarium*, and was found to accurately reflect proportions of the different species (Edel-Hermann et al., 2015; Karlsson et al., 2016). Karlsson et al. (2017a) used this metabarcoding approach to describe the *Fusarium* community in wheat grains. In one specific year of sampling of 18 fields in Sweden, they found 12 operational taxonomic units (OTUs) belonging to nine *Fusarium* species. *TEF-1α* amplicons were sequenced using 454 sequencing. However, long amplicons of the sizes obtained by these primers (550–600 bp) could for example be sequenced using PacBio SMRT sequencing. Recently, primers targeting a shorter portion of this region to match Illumina Miseq read length has been proposed (Cobo-Díaz et al., 2019). Another strategy was developed using long amplicons covering both the ITS and D1-D3 region of the large subunit sequenced on the PacBio SMRT platform, aiming to provide species-level information for *Fusarium* and information on the general fungal community (Walder et al., 2017). Walder et al. (2017) detected one OTU corresponding to *F. avenaceum/tricinctum* in wheat residues and nine *Fusarium* OTUs in maize in a field trial in Switzerland.

### Species Co-occurrences and Interactions

The species within the FHB complex might have synergistic or competitive interactions influencing their development and ability to cause disease or produce mycotoxins. Xu X.-M. et al. (2007) and Xu X. et al. (2007) studied the response of four *Fusarium* species (*F. avenaceum*, *F. culmorum*, *F. graminearum*, and *F. poae*) upon co-inoculation on wheat plants under varying temperature and humidity conditions. They found that *F. graminearum* was the most competitive species over the environmental conditions tested, and *F. poae* the least competitive. There was a general increase in the production of mycotoxins upon co-inoculation compared to single species inoculations, also in situations where the pathogen DNA was decreased. The effect is, however, not universal, and the competitive ability of species or isolates seems to be a key factor in determining the outcome in terms of mycotoxin production. More competitive species, like *F. graminearum*, may increase their mycotoxin production at the expense of that by less competitive species such as *F. culmorum* or *F. poae* (Xu X. et al., 2007). Tan et al. (2020) showed that both FHB symptoms and mycotoxin levels on wheat heads pre-inoculated with *F*. *poae* were reduced compared to inoculation with of *F*. *graminearum* alone. The authors hypothesize that inoculations with the weak pathogen *F*. *poae*, that presented early induction of salisylic and jasmonic-related defenses, could potentially explain the suppression of a subsequent *F*. *graminearum* infection.

Interactions may also occur between different isolates or chemotypes of the same *Fusarium* species. Walkowiak et al. (2015) found that co-inoculation of two chemotypes of *F. graminearum* (3-acetyldeoxynivalenol and 15-acetyldeoxynivalenol) resulted in reduced production of mycotoxins. Vaughan et al. (2020) compared single isolate inoculations to mixtures, of different isolates of *F*. *graminearum* of the same chemotype and population origin. Disease severity was found to be lower in mixed inoculations compared to single isolates, however, no consistent pattern was observed for DON contamination. The competitive ability is highly variable within species, complicating the possibility to tell whether different species (or chemotypes) vary in their ability to outcompete another species, or whether the differences are better explained at the level of isolates (Siou et al., 2015). The significance of competitive interactions for the mycotoxin accumulation highlights the importance of studies where the whole FHB species complex is considered, rather than regarding the disease and mycotoxin contamination as an interaction between a single pathogen and its host.

Under field conditions, both positive and negative correlations have been observed among *Fusarium* spp. Such correlations could be due to either similarities or differences in climatic preferences, or to interactions among species. Both Karlsson et al. (2017a) and Bernhoft et al. (2010) found that *F. culmorum* and *F. sporotrichioides* were positively correlated. Bernhoft et al. (2010) also found that *F. graminearum* was negatively correlated with *F. avenaceum, F. culmorum*, and *F. poae*, which might be an effect of its strong competitive ability when growing on wheat heads. Other negative or positive correlations between species have been reported from field investigations (Xu et al., 2008; Bernhoft et al., 2010; Karlsson et al., 2017a).

*Microdochium nivale* and *M. majus* often co-occur with *Fusarium* and can also cause head blight. Several studies have indicated negative correlations between *Fusarium* and *Microdochium* species. Nielsen et al. (2011) found that *M. nivale/majus* occurred in >90% of Danish grain samples, and grains had generally a higher biomass than grains infected with most *Fusarium* species, with the exception of *F. graminearum*. Although *M. majus* has been found to be highly abundant on lower wheat leaves, it can be almost absent from kernels where *F. graminearum* dominates (Waalwijk et al., 2004). Fungicide application decreasing the amount of *Microdochium* spp, have been found to result in increases of *Fusarium* spp. and associated mycotoxins (Simpson et al., 2001).

The *Fusarium* species patterns are variable, and it is still difficult to draw a conclusion on the general co-occurrence patterns of different *Fusarium* species. A synthesis is also made challenging by the variation in target species and methods used in the different studies. For a more comprehensive understanding of such patterns, additional field studies using metabarcoding approaches are needed, as well as controlled studies on interaction effects considering intra-species variability under variable environmental conditions.

## FHB Disease Cycle and the Cereal Microbiome

*Fusarium* fungi may interact with the cereal microbiome at different stages of the FHB disease cycle and on different plant compartments (Figure 1). The plant harbors several specific niches for microbes, in connection to soil – in the roots and rhizosphere and on aboveground plant parts such as leaves, stems and heads. In general, the aboveground microbiome is less diverse than the rhizosphere microbiome (Leach et al., 2017). Nutrients are much scarcer on aboveground tissues and microbes are more exposed to environmental stress such as drought and UV-radiation. Annual crops have to be colonized during the growing season, while the soil offers many species an opportunity to survive saprotrophically. For aboveground plant parts, soil, air, seed and other plants are important as inoculum sources (Vorholt, 2012). While in the roots and rhizosphere, it is thought that plants recruit the microbiota from the surrounding soil. There is a succession of species during the growth season, where the plant is one factor shaping the microbiome due to root exudates (Chaparro et al., 2014) and leaking of nutrients from leaves. On leaves, the succession patterns over the growing season starts with bacteria followed by yeasts and finally filamentous fungi (Kinkel, 1997). It has furthermore been shown that phyllosphere bacterial diversity decrease over the growing season and that the community become more distinct from the soil microbiome over time (Copeland et al., 2015). The plant microbiome is further influenced by the environmental conditions such as weather, soil type and agricultural management (Fierer, 2017).

### Roots, Rhizosphere, and Stem Bases

*Fusarium* fungi can be present in both seed, soil, and crop residues and act as inoculum for seedling blight, FCR and later also FHB. Root and rhizosphere microbiomes are influenced by plant species (Philippot et al., 2013). Cropping sequence may influence both the abundance of *Fusarium* inoculum and the abundance of potential antagonists present in the soil. Both will influence the health of the belowground plant parts. Borrell et al. (2016) characterized fungal communities in the roots of pulses and cereals. They found that the *Fusarium* species *F. redolens* and *F. tricinctum* were more abundant in pea roots than in wheat roots. Although pulses as preceding crop compared to wheat increased wheat productivity, no legacy effect was observed in root fungal communities of the following wheat crop. The same result was obtained by Esmaeili Taheri et al. (2016) characterizing fungal communities on durum wheat roots grown after different preceding crops. When isolates were classified into functional groups, it was found that more potential fungal antagonists, for example *Trichoderma spp.*, were present on durum wheat roots after pea than after chickpea. In contrast, potential pathogens, including *F. culmorum* and *F. acuminatum* as well as other known root pathogens of wheat, were more abundant after one of the chickpea cultivars. The abundance of both functional groups was also correlated to wheat yield. Legacy effects of the preceding crop on fungal communities in the soil and roots of the following wheat crop was demonstrated in recent metabarcoding studies (Detheridge et al., 2016; Friberg et al., 2019). Some effects of previous wheat genotype on culturable and non-culturable bacteria in wheat rhizosphere have also been reported (Donn et al., 2015). The effects of crop genotype and cropping sequence were relatively small compared to the seasonal effects, leading the authors to suggest that selection of the right sampling scale is important for studying varietal effects on the structure and function of the rhizosphere microbiome (Donn et al., 2015). That the preceding crop influences the amount of *Fusarium* inoculum in the field is already known. This is due to the quality of the plant as a host for *Fusarium*. The above-mentioned studies indicate that also the abundance of other microorganisms may be affected, these microorganisms may have antagonistic interactions with *Fusarium*. Future studies could target the importance of the microbiome in preceding crop effects on *Fusarium* disease incidence.

Similarly to roots, stem bases are also plant compartments where *Fusarium* disease may occur and infected stem bases may increase the *Fusarium* inoculum in the field. In a study characterizing fungal communities on wheat plants using tRFLP, *M. nivale* and *Oculimacula yallundae* had a high incidence on stem bases, but decreased on stems, while the opposite was true for *Davidiella* and *Cladosporium* (Grudzinska-Sterno et al., 2016). The authors found positive associations between *F. poae*, *F. avenaceum*, and *Fusarium* spp. on stems, while *Fusarium* spp. correlated negatively with *Parastagonospora nodorum* and *Zymoseptoria tritici*. On stem bases, there was a positive correlation between *F. avenaceum* and *P. nodorum*. Several yeasts were also present in relatively high incidence (Grudzinska-Sterno et al., 2016), overlapping with those identified on leaves (Karlsson et al., 2014). Gdanetz and Trail (2017) found that bacterial diversity on wheat stems was comparable to that on leaves, but substantially lower than that on roots.

### Leaves

The leaf is another potential point of interaction between *Fusarium* and the cereal microbiome. *Fusarium* species have limited ability to infect healthy leaves, but can cause symptoms in wounded leaves (Imathiu et al., 2009). However, gradual spread of *Fusarium* from stem bases, leaves and to the heads has been reported, with *Fusarium* sporulating on the leaves (Zinkernagel et al., 1997). Different cereal species harbor distinct leaf fungal communities and there is also differences between genotypes (Sapkota et al., 2015). One study on wheat found that location was more important in younger leaves, while cultivar was more important in older leaves (Sapkota et al., 2017). The mycobiome varies over the season, with Dothideomycetes increasing over plant maturity on wheat leaves (Gdanetz and Trail, 2017). Fungicide use has been reported to have relatively mild impact on the overall fungal communities on leaves (Karlsson et al., 2014; Sapkota et al., 2015). Knorr et al. (2019) showed that the total fungal abundance on leaves did not decrease after fungicide application but that relative abundance shifted in favor of yeasts, and pathogens able to infect late in the season. Köhl et al. (2007) identified only low amounts of *Fusarium* spp. on green leaves using qPCR. *M. nivale* was present on green leaves, while *F. avenaceum* was abundant on senescent leaves. Recent metabarcoding studies have not reported *Fusarium* spp. as a major part of the fungal leaf community in cereals (Karlsson et al., 2014, 2017b; Sapkota et al., 2015).

Gu et al. (2010) investigated the effect of fungicides on bacteria on wheat leaves using clone libraries and denaturing gradient gel electrophoresis (DGGE). Only γ-Proteobacteria were identified, including the *Pseudomonas*, *Buchnera*, and *Pantoea* genera. Fungicide use had an impact on bacterial communities and was associated with increased diversity. Using metabarcoding, many more phyla were identified from wheat leaves, stems and roots, Proteobacteria were identified as the most abundant phylum followed by Bacteriodetes and Firmicutes (Gdanetz and Trail, 2017).

### Heads

It is well known that cereals heads differ in susceptibility to *Fusarium* infection over the season (Yoshida et al., 2007; Tekle et al., 2012; Siou et al., 2014). Several studies have investigated the fungal communities on cereal heads or harvested grain using metabarcoding. In a study in Denmark, an OTU corresponding to *F. graminearum* and closely related species was the most abundant OTU in wheat grain, followed by *Alternaria infectoria*. Two more *Fusarium* OTUs were among the top twenty (Nicolaisen et al., 2014). The authors identified three co-occurring clusters of OTUs, one consisting of saprotrophs, one of yeasts/saprotrophs and one of wheat pathogens. A study following the fungal community on wheat heads over the season found that the total amount of fungal DNA increased during head maturation. Simultaneously, the composition changed, so that the proportion of Ascomycota increased over Basidiomycota with time. *Alternaria* and *Cladosporium* were the most prevalent OTUs. Several *Fusarium* OTUs were identified but were not among the most abundant members of the community (Hertz et al., 2016). In a study on harvested wheat grain, *Alternaria alternata* and *F. graminearum* were isolated from all samples (González et al., 2008).

On fresh barley grains, *Alternaria* and several yeasts such as *Cryptococcus* were the most abundant (Chen et al., 2016). *Fusarium* and *Alternaria* relative abundance increased from fresh grain to malts, while yeasts and *Cladosporium* decreased. Interestingly, there was a higher fungal load on barley grains, including *Fusarium*, when the crop was harvested directly compared to swathing (Chen et al., 2016). Studies on the microbiome of oats are lacking although oats can be heavily contaminated with *Fusarium* mycotoxins, and with different *Fusarium* species than those on wheat (Hofgaard et al., 2016a; Edwards, 2017).

The bacterial community on cereal heads or grains is less well-characterized. In a study of wheat kernels, the most abundant phyla were Proteobacteria, Actinobacteria, Bacteroidetes and Firmicutes (Bakker and McCormick, 2019). Minervini et al. (2015) used metabarcoding of Firmicutes to target lactic acid bacteria in durum wheat roots, leaves and spikes throughout the growing season. The authors identified six core lactic acid bacterial genera present in >98% of samples. *Lactobacillus plantarum* was present in all organs and at all growth stages as an endophyte. *L. plantarum* is also known as a promising biocontrol agent of FHB (Legrand et al., 2017).

Recent studies have characterized the structure of the cereal microbiome and the influence of different factors such as plant organ, management practices or environmental factors. Although there are significant variations, several fungal genera are repeatedly recovered from various geographical areas, suggesting that there is a core set of fungi adapted to cereals. Several ascomycetes, including *Alternaria* spp., *Epicoccum* spp., and *Cladosporium* spp. are among the most common fungi occupying the same niche as the FHB species complex on mature cereal heads and harvested grain. However, the ratio of *Fusarium* species to other fungi can vary greatly and the factors influencing this relationship are not fully understood. For instance, *Alternaria* and *Fusarium* have been found to have contrasting associations with microclimatic variables in a heterogenous wheat field, where *Fusarium* was related cooler and wetter spots while *Alternaria* abundance correlated to warmer and dryer places (Schiro et al., 2018). There is a need to closer examine the relative importance of biotic and abiotic interactions between *Fusarium* spp. and other fungal species for their success in colonizing cereal crops.

## Toward Microbiome-Based Management of FHB

There are many examples of beneficial microbial strains that can reduce plant diseases. By studying interactions between single microbial strains and pathogens, several mechanisms involved in disease suppression have been identified including, antibiosis, mycoparasitism, and competition. Beneficial microorganisms may also restrict pathogens indirectly, by affecting the plant, for instance by inducing resistance (Pieterse et al., 2014). From a microbiome perspective, these interactions become more complex and the possibility for indirect effects increases. A well-known example of disease suppression where more complex mechanisms are involved is disease suppressive soils. Disease suppression can be specific, affecting only one pathogen, or more general restricting a broad range of pathogens. It has been proposed that specific disease suppression is linked to populations of antagonistic species, while the general suppression is due to more complex communities of microorganisms (Schlatter et al., 2017). Pathogen-microbiome interactions may also have negative outcomes for plant health. Microbial species ranging from commensal to pathogenic have been shown to facilitate infection or increase disease severity (Dewey et al., 1999; Jung et al., 2018; Seybold et al., 2020). Mechanisms can also be indirect, by suppressing plant defense (Seybold et al., 2020).

There are different strategies for exploiting plant-associated microbes to suppress or limit plant pathogens. One strategy is to identify individual beneficial species and apply these in crop production formulated in biocontrol products (augmentative biocontrol) (Sundh and Goettel, 2013). For FHB, biocontrol agents inoculated at the time of flowering is an attractive alternative to chemical control as the window of protection is narrow (Gilbert and Fernando, 2004). Many studies have evaluated such biocontrol agents to limit FHB (for a review see Legrand et al. (2017). A number of fungal and bacterial strains with biocontrol effect against FHB have been identified in genera such as *Cryptococcus*, *Trichoderma*, *Clonostachys*, *Bacillus*, *Lactobacillus*, *Pseudomonas*, and *Streptomyces*. Mycoparasitism has been suggested as the mode of action for some of the fungal genera including *Trichoderma* and *Clonostachys* (Karlsson et al., 2015). While production of antifungal secondary metabolites (antibiosis) has been the proposed mechanism for many of the bacterial antagonists (Zhao et al., 2014; Palazzini et al., 2016), but also for some fungal antagonists (Rodríguez et al., 2011). Nutrient competition through iron-chelating siderophores has been implied in antagonism against *F*. *graminearum* (Pal et al., 2001). Another example is the use of choline-metabolizing biocontrol strains, since choline is a compound present in wheat anthers which stimulates hyphal growth of *F. graminearum* (Schisler et al., 2006). Induced systemic resistance has been implied in the interaction between a *Pseudomonas* strain and root-infection by *F. graminearum* in barley (Henkes et al., 2011). Although many promising biocontrol agents against FHB have been identified, so far very few products have reached the market (Legrand et al., 2017). This may be due to difficulties in achieving consistent control effects under field conditions, challenges in the formulation process, and time-consuming and expensive approval processes (O’Callaghan, 2016; Sundh and Eilenberg, 2020).

Another biocontrol strategy is to focus on the indigenous microbial community, trying to stimulate an active and diverse community, often referred to as conservation biocontrol. There are also links between the augmentative and conservation biocontrol, for example it is important to understand the interactions of biocontrol agents with the indigenous microbiome (Massart et al., 2015). Developing consortia consisting of several biocontrol agents also requires understanding of species interactions.

In the following sections we will focus on studies addressing the potential for reducing *Fusarium* inoculum in crop residues by using biotic interactions and the potential for using the cereal microbiome in strategies to limit FHB development and mycotoxin contamination.

### Interactions on Crop Residues

Due to the important role of crop residues as an inoculum source for FHB, many studies have targeted biological interactions on residues aiming to reduce survival of *Fusarium* spp. These interactions can affect both the survival of different *Fusarium* species and their ability to produce perithecia and ascospores or asexual conidia. A wide range of organisms will influence the ability of *Fusarium* spp. to survive on crop residues. Soil fauna are of importance because they feed on fungi colonizing the crop residues and directly on the residues, which enhances the decomposition process. Earthworms, especially the deep-burrowing (anecic) species also take residues from the soil surface and pull them down into their burrows, thereby contributing to the removal of straw from the soil surface (Friberg et al., 2005). All these effects make earthworms contribute to the reduction of inoculum of *Fusarium* spp. surviving on the residues. Wolfarth et al. (2011) found for example that the anecic earthworm *Lumbricus terrestris* increased the incorporation of straw and reduced the biomass of *F. culmorum* under field conditions. Smaller soil animals like nematodes also feed selectively on residue colonizing fungi. Similarly to earthworms, they can directly reduce the biomass of certain species and change the competition and succession of species during the decomposition (Friberg et al., 2005).

Crop residues left on the soil surface have a slower decomposition rate than residues buried in the soil, which enables longer survival of *Fusarium* spp. on the residues. Pereyra et al. (2004) studied the survival of *F. graminearum* on straw buried in the soil or left on the surface and found that 25% of the dry matter remained after 24 months at the soil surface, compared to 2% after burial in the soil. In addition to this effect on the decomposition of straw, they found important effects from other organisms outcompeting *F. graminearum*. While being a strong competitor on the wheat head, *F. graminearum* seems to be a relatively weak competitor during saprotrophic growth, especially during later stages of decomposition (Fernandez et al., 2008; Leplat et al., 2012). Pereyra et al. (2004) found that *F. graminearum* decreased in abundance over time, while the abundance of other *Fusarium* species increased, suggesting that several other *Fusarium* spp. initially present in the straw (*F. poae*) or found in soil (*F. oxysporum*, *F. solani*) are better at colonizing the partially decomposed plant material than *F. graminearum*. Reduced survival of *F. graminearum* has also been associated with increases in antagonistic streptomycetes in soil following soil amendments of green manure (Perez et al., 2008). Legrand et al. (2019) measured the ability of *F*. *graminearum* to grow in soils from 31 wheat fields in France. They found that bacterial richness was lower in soils conducive to *F*. *graminearum* growth, and that conducive soils were richer in iron and manganese, compared to suppressive soils. The possibility to stimulate microbial degradation or antagonism against *Fusarium* spp. through cultural practices is a research field that should be explored further.

In addition to effects on the survival of *Fusarium* spp. on decomposing crop residues, microbial interactions can have an effect on the production of spores. Special interest in this field has been on reducing perithecia formation and ascospore production by inoculation of fungi with known biological control effects on *Fusarium* species or other plant pathogens. Isolates of *Trichoderma harzianum* (several isolates), *Microsphaeropsis* sp. (isolate P130A) or *Clonostachys rosea* (isolate ACM941) applied to crop residues have been shown to reduce perithecia formation of *F. graminearum* both under laboratory and field conditions (Fernandez, 1992; Bujold et al., 2001; Inch and Gilbert, 2007, 2011; Hue et al., 2009; Schöneberg et al., 2015). The mechanism behind the suppression by *Microsphaeropsis* sp. and *C. rosea* is not well understood. Schöneberg et al. (2015) saw that co-culturing on agar gave low prediction for the ability to reduce perithecia formation. In their study, the only isolate that could suppress perithecia formation when inoculated after *F. graminearum* (*C. rosea*) gave weak suppression in co-culture assays. In the suppression by *T. harzianum*, colonization of perithecia, especially before maturation, has been observed. It is also possible that secondary metabolites produced by *T. harzianum* interferes with perithecial development and potassium uptake, thereby preventing the build-up of osmotic pressure in the perithecia (Inch and Gilbert, 2011). Application of biological control fungi to crop residues might also have an effect on the formation of conidia. Luongo et al. (2005) found that *C. rosea* suppressed the conidia formation by *F. culmorum*, *F. graminearum*, *F. proliferatum*, and *Fusarium verticillioides* on wheat straw or maize stalks under controlled conditions, but that such effects were inconsistent under field conditions.

### Suppressiveness of the Cereal Microbiome

Several studies have screened naturally occurring microorganisms on wheat for *Fusarium* suppression. Gdanetz and Trail (2017) screened endophytic fungi and bacteria in a biotest with *F. graminearum* on wheat seedlings and identified several strains reducing disease severity: *Alternaria tenuissima* and *Alternaria* sp., *F. oxysporum*, *F*. *solani* and *Fusarium* sp., *Phoma* sp. and *Penicillium reticulisporum* and *Penicillium commune*. In another example, starting with 758 isolates, 13 bacterial and fungal strains significantly reducing *F. graminearum* disease on detached wheat spikelets were identified (Comby et al., 2017), which belonged to the species *Bacillus amyloliquefaciens*, *Aureobasidium protae*, *Clonostachys rosea*, *Microdochium bolleyi*, *Phoma glomerata*, and *Sarocladium kiliense* (Comby et al., 2017). Another study isolated *Pseudomonas* bacteria from wheat leaves of which 15% showed antagonistic activity against *F*. *graminearum* or *F*. *culmorum in vitro* (Müller et al., 2016). Of the antagonists, 23% possessed the phlD gene, involved in the biosynthesis of 2,4-diacetylphloroglucinol (DAPG), an antibiotic with anti-fungal activity, indicating one possible mechanism of *Fusarium* suppression. A factor to consider is that different *Fusarium* isolates have been shown to respond very differently to antagonistic *Pseudomonas* strains (Müller et al., 2018). Most studies aim at identifying promising biocontrol agents, selecting among isolates in several steps, and do not aim to infer the overall level of suppressiveness of different plant compartments for example. This kind of comparisons would be interesting to identify cropping practices associated with a more suppressive microbiome. However, it has been reported that yeasts isolated from soil had better suppression against 16 fungal pathogens, including several *Fusarium* species, than those from the aboveground plant parts (Hilber-Bodmer et al., 2017).

As opposed to screening isolates for *Fusarium* suppression, several authors identified negative associations between *Fusarium* abundance and other microorganisms using molecular methods. Köhl et al. (2015) quantified initial abundance of eight *Fusarium* pathogens using qPCR and characterized the fungal and bacterial communities on maize stalks. After field exposure, large variation in the abundance of *Fusarium* species was observed between stalks. Several fungal and bacterial genera associated with stalks with lower *Fusarium* levels were identified including *Cryptococcus* spp., *M. bolleyi*, and several bacterial genera such as *Bacillus* and *Pseudomonas*. In another example on wheat spikes, fungal taxa *Cladosporium*, *Itersonilia*, and *Holtermanniella* had higher abundance on spikes lacking FHB symptoms (Rojas et al., 2020). A study on individual wheat kernels identified negative associations between mycotoxigenic *Fusarium* spp. and *Sphingomonas* (Bakker and McCormick, 2019). High *Fusarium* abundance was also linked to lower fungal and bacterial diversity in these two studies. Whether these observations are the result of active competition between species or an effect of environmental or plant factors is of interest to explore further.

Several studies have identified fungal species belonging to genera with many wheat pathogens as promising for biocontrol of FHB such as *Microdochium*, *Alternaria*, or *Fusarium* (Comby et al., 2017; Gdanetz and Trail, 2017). *M. bolleyi* is a common root endophyte of wheat and antagonistic against several root pathogens (Sieber and Grünig, 2006), although an effective antagonist in vitro it can also have deleterious effects in planta (Gdanetz and Trail, 2017). As was detailed before, *Alternaria* spp. are common on cereal heads, of which some are pathogenic and produce several mycotoxins (Lee et al., 2015). Effects of *Fusarium* and *Alternaria* species on each other was investigated on sterilized wheat kernels (Müller et al., 2014). *Alternaria tenuissima* grew slightly better in presence of *Fusarium* toxins, and was able to degrade both DON and ZEA. On the contrary, *F. graminearum* and *F. culmorum* could not degrade *A. tenuissima* toxins. In addition, *F. graminearum* and *F. culmorum* decreased and increased, respectively their production of ZEA in the presence of *A. tenuissima* toxins.

An interesting observation suggesting a role of competing microorganisms in FHB is that *Fusarium* infection may even be increased after fungicide application targeting foliar pathogens (Henriksen and Elen, 2005). However, studies testing the effect of competing saprotrophs on FHB in living plants have rendered variable results. Inoculation with *Alternaria*, *Botrytis* or *Cladosporium* at GS 69 (anthesis complete) before inoculation with *F. culmorum* reduced FHB severity (Liggitt et al., 1997). But in a similar experiment, when *Alternaria*, *Cladosporium* or *Microdochium* was inoculated as GS 57 (3/4 of inflorescence emerged) before *F. culmorum* inoculation FHB severity and DON concentration in grain increased (Pirgozliev et al., 2012).

Recently the importance of the microbiome for disease suppression as a community has been highlighted. For example, it was shown that a transplanted microbiome could transfer resistance to a foliar pathogen in another environment (Zahn and Amend, 2017). Another example is that leaf wash could restore pathogen resistance of cuticle mutants (Ritpitakphong et al., 2016). This kind of approaches directly manipulating the microbiome have not been explored in *Fusarium* cereal-pathosystems but could be a way forward to identify indicators for *Fusarium*-suppressive microbiomes or management strategies promoting disease suppressive indigenous communities.

Another interesting strategy is to incorporate the microbiome in breeding (Wei and Jousset, 2017). There are several examples from cereal crops demonstrating the impact of breeding on the root and rhizosphere microbiome (Alegria Terrazas et al., 2020; Kinnunen-Grubb et al., 2020). Valente et al. (2020) showed that root colonization by *Pseudomonas kilonensis*, a plant growth promoting rhizobacterium (PGPR), was higher in ancient wheat cultivars than in modern ones under gnotobiotic conditions. When a smaller set of cultivars were tested in non-sterile soil, there was no difference in colonization, but the same cultivars that had higher colonization in gnotobiotic conditions had enhanced root growth under water and nutrient stress in non-sterile soil. The authors hypothesize that dwarfism and the associated reduction in response to gibberellic acid in modern cultivars may be a trait affecting the interaction with PGPR. Identifying plant traits that are associated with the recruitment of a *Fusarium*-suppressive microbiome would be highly relevant. These traits could then be used as breeding targets. Beneficial microbes may also be introduced during reproduction so that they are vertically transmitted via the seed to coming generations (Mitter et al., 2017).

## Conclusion and Outlook

It is clear that both interactions between the species responsible for FHB, and interactions with other members of the plant microbiome play an important role in disease outbreaks and for mycotoxin accumulation in cereals. The ability to handle these problems would benefit greatly from a more thorough and detailed understanding about these interactions, and how such information can be implemented in prediction models and disease control programs. Today, we lack information about many key questions regarding how and when in the disease cycle of FHB these interactions can be utilized, to produce healthy crops with no or low mycotoxin contamination levels.

From our review we have identified several research needs. Much of the literature on FHB has a focus on *F. graminearum* on wheat, while problems are caused by several other species and in all cereal crops. There are examples that different *Fusarium* species may respond differently to control methods (Pirgozliev et al., 2003; Vogelgsang et al., 2008). Several regions have also experienced shifts in predominant species (Valverde-Bogantes et al., 2019) and the legislation will likely cover more toxins produced by other species in the future. *F. graminearum* is interacting with other *Fusarium* species with differences in interactions depending on the crop species as well as on environmental conditions. New methods have been developed that will be highly valuable to assess the full diversity of the FHB species complex in different environments. For example, metabarcoding approaches (Table 2) are powerful tools to study co-occurrence patterns at different scales.

Observations of competitive interactions or negative associations between different *Fusarium* species and between *Fusarium* species and other members of the cereal microbiome are common. For instance, negative associations between *Fusarium* and other pathogenic fungi occupying the same niche such as *Microdochium* sp. and *Alternaria* sp. have been observed (Nielsen et al., 2011; Schiro et al., 2018). This could be due to contrasting environmental preferences or antagonistic or competitive interactions. *Fusarium*-antagonistic members in these taxa have indeed been isolated from cereals (Comby et al., 2017; Gdanetz and Trail, 2017). Mycotoxins may also play a role in species interactions and their ecological role should be further explored (Venkatesh and Keller, 2019). A better understanding of the mechanisms involved in species interactions and how these are influenced by the environmental conditions, is key to make accurate predictions of disease development and mycotoxin production. It may also give clues to which traits biocontrol agents should possess and under which conditions they should be applied in order to be most successful.

Several studies have in recent years evaluated the variation of the cereal microbiome structure over different plant organs, environmental conditions and management strategies (Donn et al., 2015; Gdanetz and Trail, 2017; Knorr et al., 2019). Cereal heads are relatively species poor compared to for instance soil or roots (Nicolaisen et al., 2014; Hertz et al., 2016; Friberg et al., 2019), and the relative competitive ability of *Fusarium* fungi differ among these microenvironments (Leplat et al., 2012). Disease control methods may be directed towards different stages of the FHB disease cycle such as reducing *Fusarium* survival in soil and in crop residues and limiting infection, growth or mycotoxin production on cereal heads. Microbiome-based strategies to limit FHB are still to be achieved and future studies are needed to identify the characteristics of cereal microbiomes linked to *Fusarium* suppression. Manipulating microbiomes or creating synthetic communities could be an approach to go from observations to a more mechanistic understanding (Vorholt et al., 2017).

Recent technological development has revolutionized our ability to characterize plant associated microbiomes on one hand, and to get in-depth insights into interactions between plant pathogens and their hosts or antagonists on the other hand. But there is a gap in knowledge between these two research fields that needs to be filled in order to use the potential of microbiomes in plant disease control, either through adding biocontrol agents to the crop, or through conservation biological control, where beneficial microbial populations are stimulated through carefully selected cropping practices or in resistance breeding strategies.

## Author Contributions

All authors planned the study, wrote the manuscript, and approved the final version of the manuscript.

## Conflict of Interest

The authors declare that the research was conducted in the absence of any commercial or financial relationships that could be construed as a potential conflict of interest.
